# Chromatin and siRNA pathways cooperate to maintain DNA methylation of small transposable elements in *Arabidopsis*

**DOI:** 10.1186/gb-2005-6-11-r90

**Published:** 2005-10-19

**Authors:** Robert K Tran, Daniel Zilberman, Cecilia de Bustos, Renata F Ditt, Jorja G Henikoff, Anders M Lindroth, Jeffrey Delrow, Tom Boyle, Samson Kwong, Terri D Bryson, Steven E Jacobsen, Steven Henikoff

**Affiliations:** 1 Division of Basic Sciences, Fred Hutchinson Cancer Research Center, 1100 Fairview Ave N, Seattle, WA 98109, USA; 2 Howard Hughes Medical Institute, Fred Hutchinson Cancer Research Center, 1100 Fairview Ave N, Seattle, WA 98109, USA; 3 Department of Genetics and Pathology, Rudbeck Laboratory, Uppsala University, Uppsala, 751 85 Sweden; 4 Department of Molecular, Cell and Developmental Biology, University of California Los Angeles, Los Angeles, CA 90095, USA

## Abstract

**Background:**

DNA methylation occurs at preferred sites in eukaryotes. In *Arabidopsis*, DNA cytosine methylation is maintained by three subfamilies of methyltransferases with distinct substrate specificities and different modes of action. Targeting of cytosine methylation at selected loci has been found to sometimes involve histone H3 methylation and small interfering (si)RNAs. However, the relationship between different cytosine methylation pathways and their preferred targets is not known.

**Results:**

We used a microarray-based profiling method to explore the involvement of *Arabidopsis *CMT3 and DRM DNA methyltransferases, a histone H3 lysine-9 methyltransferase (KYP) and an Argonaute-related siRNA silencing component (AGO4) in methylating target loci. We found that KYP targets are also CMT3 targets, suggesting that histone methylation maintains CNG methylation genome-wide. CMT3 and KYP targets show similar proximal distributions that correspond to the overall distribution of transposable elements of all types, whereas DRM targets are distributed more distally along the chromosome. We find an inverse relationship between element size and loss of methylation in *ago4 *and *drm *mutants.

**Conclusion:**

We conclude that the targets of both DNA methylation and histone H3K9 methylation pathways are transposable elements genome-wide, irrespective of element type and position. Our findings also suggest that RNA-directed DNA methylation is required to silence isolated elements that may be too small to be maintained in a silent state by a chromatin-based mechanism alone. Thus, parallel pathways would be needed to maintain silencing of transposable elements.

## Abstract 2

Microarray-based profiling of the involvement of two DNA methyltransferases (CMT3 and DRM), a histone H3 lysine-9 methyltransferase (KYP) and an Argonaute-related siRNA silencing component (AGO4) in methylating target loci in *Arabidopsis* reveals that transposable elements are the targets of both DNA methylation and histone H3K9 methylation pathways, irrespective of element type and position.

## Background

DNA cytosine methylation is an ancient process, found in both prokaryotes and eukaryotes, and catalyzed by a single family of methyltransferases [1]. In prokaryotes, cytosine-5 methyltransferases protect target sites from cleavage by partner restriction endonucleases, but in eukaryotes, the function of DNA methylation is less clear. In organisms that retain DNA methylation, including plants, most animals and some fungi, it has been speculated that DNA methylation provides genomic immunity against mobile elements [2,3]. This hypothesis has been difficult to test in vertebrates, because most CG dinucleotides are heavily methylated in both genic and intergenic regions [4]. In fungi and plants, however, the localized nature of DNA methylation makes it possible to identify sequences that are targeted for DNA methylation. For example, in *Neurospora*, DNA methylation occurs at repeated sequences that are targeted for point mutation [5]. In plants, transposable elements are heavily methylated relative to genic regions, suggesting that the silencing that accompanies DNA methylation is a means of defending against transposition [3,6,7]. An additional form of DNA methylation is found in the model plant *Arabidopsis*, where short dense CG methylation clusters are occasionally found in genic regions that are otherwise devoid of methylation [8].

Although many DNA methylation targets are known, it has been unclear how these sites are recognized by DNA methyltransferases. The Dnmt3 subfamily of DNA methyltransferases, which includes *Arabidopsis *DRM1 and DRM2, can methylate *de novo *[9], but there are no known sequences in common among target sites. Recent work in *Arabidopsis *has implicated the small interfering (si)RNA machinery in targeting *de novo *methylation [10-12], and a large number of transposon-directed siRNAs have been sequenced [13,14]; however, the mechanism by which siRNA production leads to *de novo *DNA methylation is not known. Another open question is how some forms of DNA methylation are maintained during rounds of cell division. In the case of CG sites, a member of the Dnmt1 subfamily of DNA methyltransferases maintains methylation by specifically methylating hemi-methylated sites behind the replication fork [15], but in cases of non-CG methylation, there does not appear to be a comparable reaction. Non-CG methylation in *Neurospora *is maintained by the action of a histone H3 lysine-9 (H3K9) methyltransferase [5], so the successive action of a histone methyltransferase and a DNA methyltransferase suffices to maintain methylation indefinitely. A similar maintenance mechanism occurs for CNG sites in *Arabidopsis*, where the KRYPTONITE (KYP = SUVH4) H3K9 methyltransferase directs methylation by the CHROMOMETHYLASE3 (CMT3) CNG methyltransferase [16,17]. These findings have led to a general model whereby siRNAs direct *de novo *methylation by DRM1 and DRM2, CG sites are maintained by the Dnmt1 ortholog, MET1, and CNG sites are maintained by the successive action of KYP and CMT3 [10,18].

These insights into DNA methylation mechanisms were obtained using sensitive reporter systems chosen because they display striking epigenetic silencing phenotypes [18]. As a result, they were not designed to reveal the spectrum of target sites acted upon by these various DNA methylation pathways. An alternative approach is to look at large numbers of sites for changes in methylation levels when mutations in various components of epigenetic silencing are introduced. In previous work, we used a microarray-based method for profiling methylation patterns to detect changes that occur in a *cmt3 *mutant line [7]. This analysis revealed hypomethylation of a subset of randomly chosen sites. This subset was enriched in transposon-derived sequences, consistent with DNA methylation playing a role in genome defense against transposable elements [7,19]. The small scale of the analysis did not allow us, however, to determine whether there are preferences for different types or locations of elements, as has been suggested for CMT3 [20].

Here we present a large-scale analysis of methylation patterns in mutants that are involved in CNG methylation. We found that CMT3 and KYP targets are transposons of all types and show a distribution along the chromosomes that is similar to that of the bulk of elements in the genome. In contrast, we found relatively few DRM and AGO4 targets scattered throughout the chromosomes, and these are significantly enriched in small isolated transposon-derived sequences. Our findings suggest a special role for RNA-directed DNA methylation in silencing mobile elements that are scattered along chromosome arms.

## Results

### Profiling of CNG methylation

To profile methylation patterns, DNA samples are treated with a methylation-sensitive restriction endonuclease and are size-fractionated by sucrose gradient centrifugation [7]. The low molecular weight fraction is collected and labeled with either of two fluorescent dyes, such that two samples can be compared by standard microarray analysis. If one sample is derived from a mutant in which methylation is reduced, then affected sites will be more frequently cleaved by the restriction endonuclease relative to wild-type. When cleavage results in an assayed fragment sedimenting faster than the 2.5 kb cutoff used in the fractionation, then there will be a stronger signal for mutant than wild-type. Conversely, if the mutant causes hypermethylation of a site, then the wild-type signal will be higher than the mutant signal. In this way, we can detect whether or not changes occur in methylation patterns from the ratio of the two dye signals, scoring as positive targets only those that are statistically significant based on repeated measurements from different biological samples [21]. Positive targets can be scored as either hypomethylated or hypermethylated.

In our original methylation profiling study, we assayed PCR-amplified fragments from loci known or suspected to be targets of DNA methylation and also PCR-amplified single-copy approximately 700 base pair (bp) fragments chosen at random from the *Arabidopsis *genome sequence, 360 in all [7]. In a subsequent study, we increased the size of this array to include 597 randomly chosen loci [8]. In the present study, we have used this 'random-PCR' array, as well as a 'gene-oligo' array consisting of 26,090 oligonucleotides (70-mers) representing essentially all annotated *Arabidopsis *genes [22], of which 10% (2,633 of 26,090) are identified as containing transposons and repetitive elements detectable by RepBase [23] and RECON [24] analysis (unpublished observations). The gene-oligo array thus samples both distal and transposon-rich pericentric chromatin regions of the genome.

To detect CNG methylation, we used the *Msp*I restriction endonuclease to digest DNA samples from five mutant lines: *cmt3*, *kyp*, *ago4*, *drm1/2 *(double mutant) and *cmt3 drm1/2 *(triple mutant), each paired with its wild-type parental line. Using the random-PCR array, we detected five loci as hypomethylated in the *cmt3 *mutant background; these represent single-copy targets of CMT3 that are methylated on the first C of one or more CCGG sites, a modification that blocks *Msp*I digestion.

**Figure 1:**
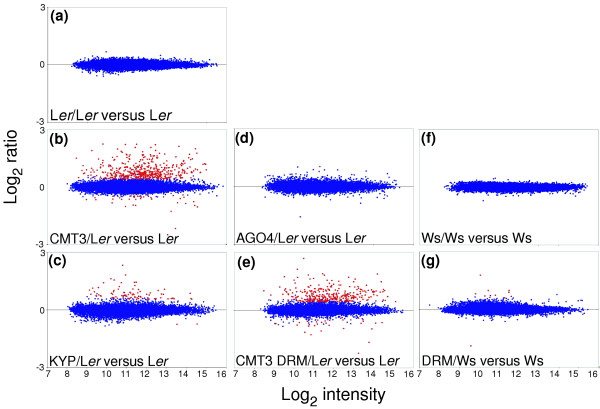
Raw data plots for the gene-oligo array. For each genotype pair, the average log_2_(exp/ref) ratio is plotted versus the corresponding average log_2 _fluorescent intensity. Each plot contains the results of six array measurements, that is dye-reversed measurements on three biological replicates. All data were lowess normalized as described in the Materials and methods section. Red dots represent statistically significant target loci, where those with positive log ratios indicate hypomethylation and those with negative log ratios indicate hypermethylation. Blue dots represent the rest of the loci.

**Figure 2:**
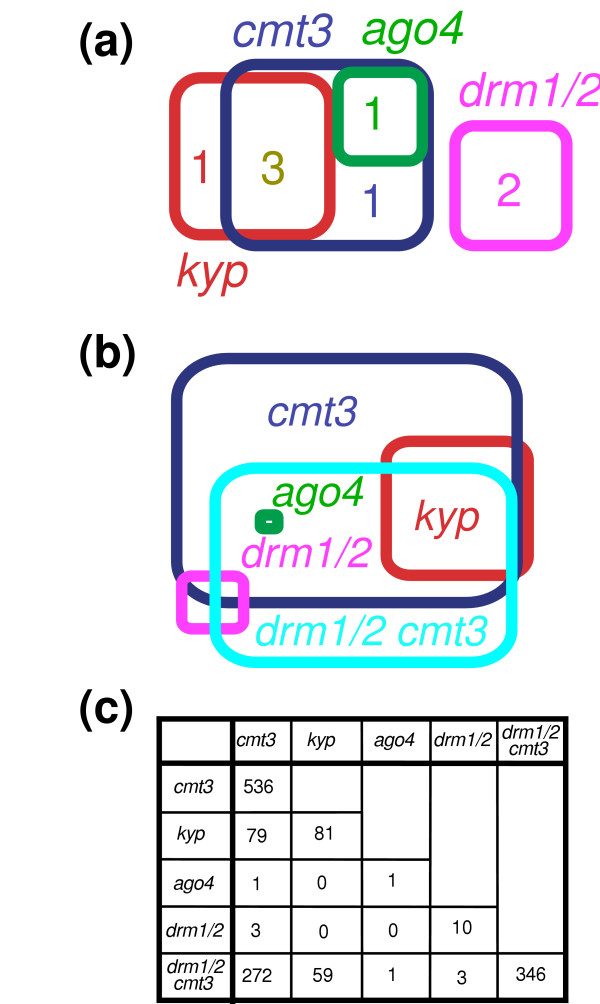
CNG methylation targets of epigenetic silencing components. **(a) **Venn diagram summaries of positive loci using random-PCR arrays in *cmt3*, *kyp*, *drm1/2 *and *ago4 *mutant backgrounds. Loci were scored as positive if methylation was significantly changed in the indicated mutant relative to the L*er *wild-type background. **(b) **Venn diagram summaries of positive loci using gene-oligo arrays, where *cmt3*, *kyp*, *drm1/2 *and *ago4 *were in a L*er *(*clk-st*) and *crm3 drm1/2 *was in a Ws wild-type background. Gene-oligo and random-PCR datasets of targets are available with a graphical interface for browsing and for downloading [25]. **(c) **Table showing the number of positives and overlaps for each mutant class. Mutants are color coded for clarity in the Venn diagrams.

We then asked whether any of these methyl-CNG positive loci were also detected as targets of other proteins assayed in this way. KYP yielded four (hypomethylated) targets from among the 597 randomly chosen single-copy loci, of which three were the same as the CMT3 targets (Table 1). Using the gene-oligo array, we detected 536 CMT3 targets (498 hypomethylated and 38 hypermethylated in *cmt3*; Figure 1a,b), and 81 KYP targets (79 hypomethylated and 2 hypermethylated in *kyp*; Figure 1c), of which 79 were also CMT3 targets (Figure 2) [25]. This nearly complete overlap shows that the interplay between CMT3 and KYP found for sensitive reporter loci [16,17] is also true for at least a large fraction of CMT3 targets genome-wide. All CMT3 or KYP positives on the random-PCR array and >90% of the positives on the gene-oligo array were hypomethylated in the mutant, as would be expected if these enzymes work in tandem to maintain CNG methylation. The close agreement between two very different array platforms [26] provides a high degree of confidence in our conclusions.

AGO4 yielded one hypomethylated target locus that was also a CMT3 target in the gene-oligo array and one (hypomethylated) target in the random-PCR array (Figures 1d and 2). In addition, we detected two targets of DRM1/2 in the random-PCR array (Table 1) and ten DRM1/2 targets (nine hypomethylated and one hypermethylated in *drm1/2*; Figure 1f,g) in the gene-oligo array (Figure 2). The low number of AGO4 and DRM1/2 targets relative to the high number of CMT3 and KYP targets on the gene-oligo array suggests that AGO4 and DRM1/2 play only a minor role in maintaining CNG methylation throughout the genome. We also assayed a triple mutant combination of *cmt3 drm1/2 *and observed extensive overlap for both the random-PCR array (Table 1) and the gene-oligo array (313 hypomethylated and 33 hypermethylated in *cmt3 drm1/2*; Figures 1e and 2).

### CMT3, KYP and DRM target transposable elements

In our original study, we noticed that all four randomly chosen loci that were positive for CMT3 represent transposable elements [7]. This correspondence was especially striking considering that the loci were chosen to be single-copy in the genome, so that these represent the rarest class of transposable elements. This conclusion is confirmed in the present study. For the purposes of our analysis, we considered only loci where methylation blockage of a single site could cause a fragment to sediment more rapidly than 2.5 kb, thus resulting in its exclusion from the DNA used as probe (see Materials and methods and [8]). By this criterion, only a subset of restriction sites overlapped by a repeat or transposon would be scored as affected by the mutation. Nevertheless, we found a preponderance of transposable elements in this class for CMT3 (Table 2). Likewise, three of the four single-copy targets of KYP were scored as transposable elements. This preference for transposable elements in CMT3 targets was amply confirmed on the gene-oligo array, with 63% (104/164) of loci with sites falling within transposons, compared with only 13% (907/7,032) for all loci on the array. These 104 elements include long terminal repeat (LTR), long interspersed element (LINE) and short interspersed element (SINE) retrotransposons, DNA transposons and helitrons. KYP showed a similar preference to CMT3 with 68% (26/38) of loci falling within transposons. We conclude that transposable elements of all types are by far the predominant target of the CMT3-KYP system. We also found a preference of DRM1/2 for transposable elements (Table 2).

**Figure 3:**
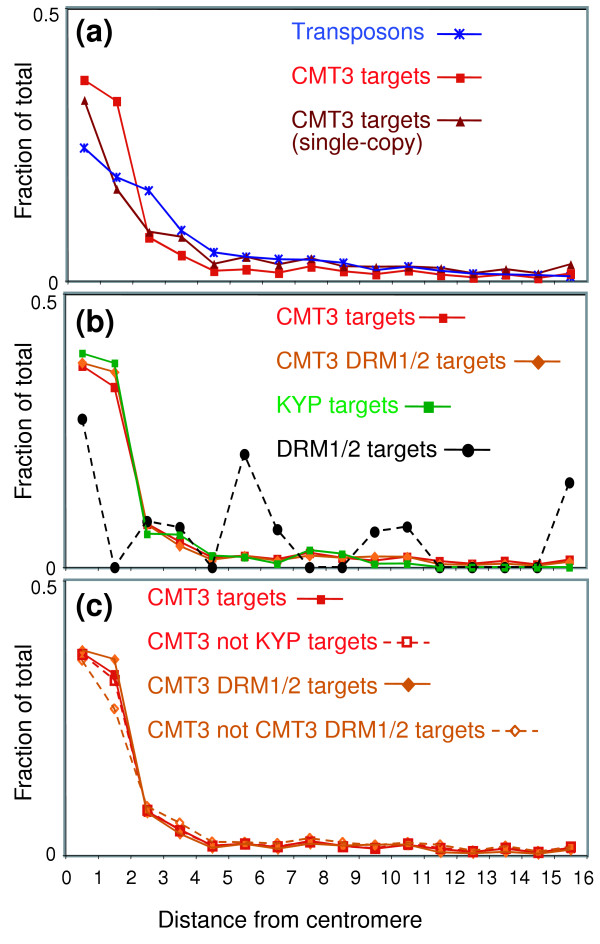
Location of transposable elements, CMT3, KYP and DRM1/2 targets along chromosome arms. **(a) **Transposable elements and CMT3 targets. **(b) **Comparison of KYP, DRM1/2 and CMT3 DRM1/2 targets to CMT3 targets. **(c) **Comparison of all CMT3 targets to the subset of CMT3 targets that are not also KYP targets, and comparison of CMT3 targets to the subset that are not also CMT3 DRM1/2 targets. To map repeats relative to the centromere, Repbase library sequences were searched using BLASTN with default Repbase parameters against TIGR Release 5 of the *Arabidopsis *genome sequence. All CMT3 targets, single-copy CMT3 targets and DRM1/2 targets from the gene-oligo array (Figure 2b) were also mapped on the same scale. The fraction of the total number of hits within each 1 Mb bin is shown. To compensate for differences in oligo abundance on the array, bins were normalized by dividing each raw fraction by the fraction of oligos in the bin.

If the targets of CMT3 result from a general preference of these DNA methylation pathways for transposable elements, then we would predict that the distribution of targets would approximately correspond to that of transposable elements along the chromosome. We tested this by comparing the distribution of target distances for CMT3 from the centromeric gap to the locations of repetitive elements. We searched the Repbase library of consensus sequences [23] against the *Arabidopsis *genomic sequence to determine the distribution of repeats. Most transposable elements in *Arabidopsis *are located near the centromeric gap, gradually decreasing in abundance towards the telomere (Figure 3a). Similarly, CMT3 targets, whether repetitive or single-copy, are highly abundant close to the centromeric gap, decreasing as one moves distally along the chromosome arms.

We wondered whether the KYP and DRM1/2 targets also showed a transposon-like distribution. This would be expected if KYP and DRM1/2 targets mostly comprise a representative sample of CMT3 targets. Indeed, the KYP target distribution along the chromosome nearly superimposes over that for CMT3 (Figure 3b), which together with the nearly complete inclusion of KYP targets within the set of CMT3 targets, indicates that KYP and CMT3 have the same target preferences. In contrast, DRM1/2 targets are scattered throughout the chromosome arms, with only one of ten targets in the most proximal 2 Mb where about half of the CMT3 targets are found; this difference in the distribution of elements is statistically significant (*p* = 0.013, Fisher's exact test). We conclude that CMT3 and KYP target transposable elements in general, whereas DRM1/2 is required primarily at elements that are distally located along the chromosome.

Our conclusion that DRM1/2 targets are distinct from CMT3 targets is unlikely to have resulted from false positives in the DRM1/2 dataset. As previously mentioned, DRM1/2 targets are enriched in transposons. In addition, the close correspondence between CMT3 and KYP distributions, even considering just the 85% of CMT3 targets that are not KYP targets (Figure 3c), implies that the cutoff criteria used for target detection were very conservative. As indicated below, it appears that the large majority of KYP targets were simply too weak relative to CMT3 targets to be detected in the context of a whole-genome analysis. Furthermore, the CMT3 DRM1/2 dataset provides an independent test of the stringency of our cutoff criteria, because we would expect it to include all of the CMT3 targets; but it actually is a smaller set that only partially overlaps. This partial overlap is evidently not attributable to false positives in both datasets, because the distributions of CMT3 and CMT3 DRM1/2 targets essentially superimpose (Figure 3c), even considering just the 21% of CMT3 targets that are not CMT3 DRM1/2 targets. This indicates that the small number of DRM1/2 targets results from strict cutoff criteria that identify a subset of truly affected loci.

### Bisulfite sequencing of CNG methylation targets

**Figure 4:**
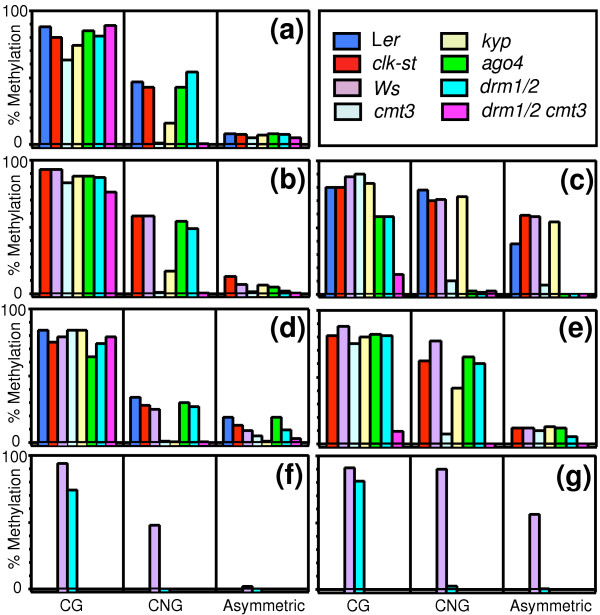
Methylation occupancies of selected target loci determined by bisulfite sequencing. Elements are: **(a) **Mu-PCR (locus 4:1813417-1814107 on random-PCR array hypomethylated in *cmt3*, *kyp *and *cmt3 drm1/2*); **(b) **229-R1 (left side of A000229 on gene-oligo array hypomethylated in *cmt3 *and *drm1/2*); **(c) **229-R2 (right side of A000229); **(d) **Mu-4802 (A004802 hypomethylated in *cmt3*, *kyp *and *cmt3 drm1/2*); **(e) **TA11-4217 (A004217 hypomethylated in *cmt3*); **(f) **SINE3-5300 (A005300); **(g) **SINE3-11193 (A011193 hypomethylated in *drm1/2*). Wild-type lines are L*er*, *clk-st *(parental line of *cmt3*, *kyp*, and *ago4 *derived from L*er*) and Ws (parental line of *drm1/2*). See Table 3 for details.

To confirm and quantify the array results, we performed bisulfite sequencing on a selection of target sites. We chose one positive example from the random-PCR array and five from the gene-oligo array. For locus 4:1813417-1814107 (Mu-PCR), detected as a target of CMT3, KYP and CMT3 DRM1/2 on the random-PCR array, wild-type methylation levels averaged 88% for the 11 CG sites and 47% for the 10 CNG sites assayed by bisulfite sequencing (Figure 4a; Table 3). In the *cmt3 *mutant background, the average level dropped to 63% for CG and to 1% for CNG methylation. This drastic decrease in CNG methylation is as expected considering that CMT3 is known to be responsible for nearly all of this modification at selected loci [27,28]. In a *kyp *mutant background, the average level dropped to 74% for CG and to 16% for CNG methylation. In this fragment, methylation of a single *Msp*I site would account for a change in fragment size and its differential fractionation prior to microarray analysis. Remarkably, the *kyp*-induced decrease in methylation at the *Msp*I site itself was only about one third (from 11 to 7 methyl-Cs of the 19 determined for this site; Table 3), confirming that methylation profiling on the random-PCR array is capable of detecting an intermediate drop in methylation levels.

Bisulfite sequencing of targets detected on the gene-oligo arrays confirmed that the positives detected on these arrays indeed reflect changes in the degree of methylation. For example, locus A000229 was detected as hypomethylated in both *cmt3 *and *drm1/2 *mutants, and bisulfite sequencing shows a reduction of methylation at one flanking *Msp*I site in *cmt3 *and at the other flanking *Msp*I site in *drm1/2 *(Figure 4b,c; Table 3). Interestingly, a reduction in methylation at the first *Msp*I site was also seen in *kyp*. This partial loss of CNG methylation in *kyp *that was not detected on the gene-oligo array could account for the low fraction of CMT3 targets that are also targets of KYP on this array (Figure 2b,c). Of the three other loci examined in *cmt3 *and *kyp *mutants, all showed a major loss of CNG methylation in *cmt3*, one showed a major loss of CNG methylation in *kyp*, one showed a minor loss of CNG methylation, and one showed no loss (Figure 4c–e). This consistently strong effect of *cmt3 *and variable effect of *kyp *on CNG methylation at unselected sites is in agreement with studies of *cmt3 *and *kyp/suvh4 *mutants at particular loci [16,17,19,27,28].

An unexpected finding was that CG methylation levels dropped five- to tenfold at two loci when both classes of *de novo*/CNG methyltransferases were absent (*cmt3 drm1/2 *in Figure 4c,e). This effect might be caused by an occasional failure of the MET1 CG maintenance methyltransferase, leading to a dependence on methyltransferases that do not require a hemi-methylated substrate [29,30].

### Methylation of small transposable elements is dependent on DRM1/2 and AGO4

We wondered whether there is an inherent difference between transposons that require DRM1/2 for methylation and those that do not. Bisulfite sequencing revealed major losses of CNG methylation in *drm1/2 *mutants at three loci: A000229 and two loci corresponding to SINE3 elements (Figure 4c,e,f). The approximately 160 bp size of these SINE3 targets of DRM1/2 contrasts with the >5 kb size of the three loci that were not affected by *drm1/2*. Only one of these SINE3 elements is present in the parental strain of *ago4 *(L*er*), and this showed a major drop in CNG methylation, whereas all three large elements that were unaffected by *drm1/2 *were also unaffected by *ago4*. Taken together, our results are consistent with the possibility that DRM1/2 and AGO4 are required to maintain DNA methylation at small, but not large transposable elements. The small size and low abundance of DRM1/2 and AGO4 targets might explain why so few of them were detected relative to CMT3 and KYP targets.

**Figure 5:**
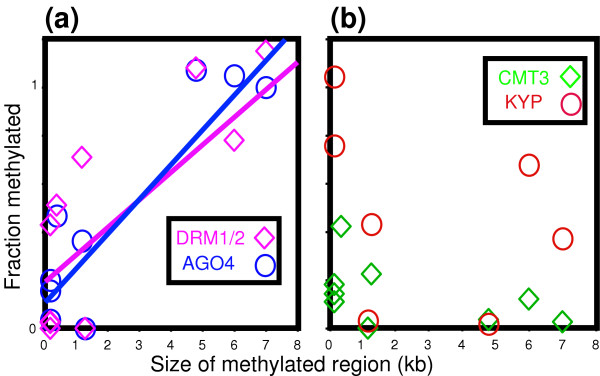
Methylation by DRM1/2 and AGO4 is associated with the size of their targets. **(a) **The loss of methylation for each locus is calculated from the reduction seen in *drm1/2 *and *ago4 *when measured by bisulfite sequencing (*drm1/2*: correlation coefficient r = 0.82, *p* < 0.003; *ago4*: r = 0.90, *p* = 0.0002). The fraction methylated is the ratio of mutant to wild-type percentages listed in Table 3. Regression lines are shown for clarity. **(b) **A similar comparison of CMT3 and KYP reveals no significant associations (*cmt3*: r = -0.48, *p* = 0.2; *kyp*: r = -0.32, *p* = 0.5), so no regression lines are shown. The comparisons include data reported in this study supplemented with previously published data for other loci [11,12].

To determine the generality of our observations on DRM1/2 and AGO4 targets, we included bisulfite sequencing data from previous studies [11,12] with our own in comparisons of levels of methylation reduction in mutants. *drm1/2 *and *ago4 *mutants show strongly correlated CNG methylation reductions (r = 0.91, *p* = 0.0002). In addition, methylation reduction shows a clear association with element size for both *ago4 *and *drm1/2 *(Figure 5a), with small elements preferentially affected. No significant associations with element size were seen for either *cmt3 *or *kyp *(Figure 5b). Methylation reduction is not attributable to differences in element type, because heterogeneous classes of sequences were found in both size classes, with DNA transposons and LINE elements in the large size class and SINEs, tandem and inverted repeats and genes in the small size class. Methylation reduction is also not attributable to differences in element abundance, because no association is seen between changes in methylation and the estimated copy number of elements in the genome (data not shown). It thus appears that AGO4 and DRM1/2 work together to maintain DNA methylation and silencing of small elements.

## Discussion

We have used DNA methylation profiling to assay the effects of mutations in *Arabidopsis *genes that have been implicated in gene silencing and epigenetic inheritance. This has led to the identification of common targets of a DNA methyltransferase and a histone modifying enzyme. The original reports of connections between these two silencing paradigms were major breakthroughs in the epigenetics field, and we have shown that this connection is widespread and not confined to a few selected and unusual loci. We also have demonstrated that the targets of both DNA methylation and histone H3K9 methylation pathways are transposable elements, irrespective of element type and position. Furthermore, we have shown that the *de novo *methylation pathway targets a selected subset of elements, and we provide data suggesting that short elements are preferentially dependent on an RNAi-mediated *de novo *methylation pathway.

We have found that nearly all CNG targets of the KYP (SUVH4) H3K9 methyltransferase are also CMT3 targets. We attribute the detection of only 79 KYP targets from among the 536 CMT3 targets to the limited sensitivity of our large-scale profiling assay, where stringent criteria are needed to eliminate false positives among the 26,090 loci scored. In support of this interpretation, bisulfite sequencing revealed a consistent partial loss of CNG methylation in *kyp *mutants. This stronger effect of CMT3 than KYP is consistent with previous work showing that CMT3-dependent DNA methylation at the *SUP *and *PAI2 *loci requires KYP (SUVH4), whereas DNA methylation of the inverted repeat *PAI1-PAI4 *locus does not [16,17]. This is also consistent with the finding that three transposons showed a greater loss of CNG methylation in *cmt3 *than in *kyp *mutants [20]. A possible reason for the variable effect of *kyp *is that other histone methyltransferases function in this capacity, and there are about a dozen *kyp *homologues in the *Arabidopsis *genome [31].

In the case of mutants in *AGO4*, a member of the Argonaute family of RISC complex components, only two sites of CNG hypomethylation were seen; this is not unexpected insofar as studies of the *SUP *locus showed weak effects of *ago4 *mutants relative to *cmt3 *and *kyp *mutants [12]. Similarly, mutations in the *drm1/drm2 de novo *methyltransferases affected only a few loci in our assay, consistent with evidence that CNG methylation is maintained primarily by the CMT3-KYP pathway [6].

As in our original methylation profiling study, we found that transposons were primary targets of CMT3 [7], and the inclusion of KYP and DRM1/2 extends this conclusion to two of the three major pathways for DNA methylation in *Arabidopsis*. Furthermore, the use of a gene-oligo array that samples most of the sequenced genome thoroughly confirms that the targets are repeats of all types, including LTR and non-LTR retrotransposons, helitrons, and MuDR and other classes of DNA transposons, and not simply a limited sample of common elements.

The use of a comprehensive gene array also allowed us to detect target differences that were not apparent from studies of single loci. In particular, we detected a preferential dependence of distally located elements on DRM1/2. It is possible that this preferential dependence results simply from the elements' distal location. If so, then we would expect to find that distal elements in general would show dependence on DRM1/2. However, two elements that were chosen for bisulfite sequencing because of their distal location (one Mu-4802 and the other TA11-4217) showed no significant loss of CNG methylation in *drm1/2*. Therefore, it appears that some property other than distal location *per se *is responsible for DRM1/2 dependence.

It is possible that DRM1/2 preferentially targets elements with corresponding siRNAs. This is suggested by the strong correlation between the degree of methylation loss in *drm1/2 *and in *ago4*, a siRNA-mediated silencing component. Indeed, most of the sequences included in our analysis that depend on DRM1/2 or AGO4 have corresponding siRNAs [13,14,19,32,33]. However, transposable elements of all types have corresponding siRNAs [13,14], indicating that siRNAs, and by inference DRM1/2 and AGO4, target transposable elements in general. Therefore, some other feature must determine whether the siRNA pathway is required to maintain DNA methylation of distal elements.

Our finding that element size is strongly associated with the degree of DRM1/2- and AGO4-dependent methylation provides a rationale for the distal location preference. Most transposable elements tend to cluster in pericentric regions in plants and animals, leading to large silent heterochromatic blocks, whereas elements that insert distally tend to be isolated. Small isolated elements might be more difficult to silence than large clustered elements [34-36], and mobile elements that are not silenced can damage the genome by replicative transposition [37]. In *Arabidopsis*, SINEs show a distribution along the chromosome [38] that is not unlike the distal distribution that we report for DRM1/2 targets. Therefore, the preferential dependence of small distal elements on DRM1/2 and AGO4 might reflect an adaptation to defend against SINEs, which would otherwise escape silencing by the chromatin-based CMT3-KYP machinery. The dependence of small elements on the DRM1/2-AGO4 pathway for DNA methylation provides support for the hypothesis that siRNA-mediated methylation reinforces unstable silencing of such elements [39].

## Materials and methods

### Sample preparations

*Arabidopsis thaliana *mutants were previously described [10,12]. To control for background variability, lines were constructed by backcrossing parental mutant lines with either L*er *(for *cmt3*, *kyp*, *ago4 *and *drm1 drm2 cmt3*) or *Ws *(for *drm1 drm2*), which served as the corresponding wild-type controls. Whole 5 week old plants were used to prepare genomic DNA using the CTAB extraction method [40]. After ethanol precipitation, DNA samples were treated with DNase-free ribonuclease (Roche, Indianapolis, IN, USA) and precipitated by addition of 3 M sodium acetate and ethanol, then pelleted by centrifugation and air-dried. Bisulfite treatment of DNA, cloning into a Topo TA vector (Invitrogen, San Diego, CA, USA) and DNA sequencing were performed as described [27]. Primer sets are listed in Table 4.

### Microarray construction

Primer selection, amplification and spotting in duplicate onto glass slides have been described in our original methylation profiling study [7]. The random-PCR array in the present study consisted of 960 loci of which 597 were randomly chosen approximately 700 bp single-copy loci and 363 were selected control loci of different lengths [8]. The 597 loci were selected as random non-overlapping 1 kb single-copy fragments from a non-redundant database consisting of contigs representing *A. thaliana *chromosomes 2 and 4 taken from a December 1999 version of the *A. thaliana *TIGR assembly [41]. Chromosomes 1, 3 and 5 were pieced together from the *A. thaliana *genome project clone table from an August 2000 version of the *A. thaliana *TIGR assembly. Most of the selected loci were amplified from segments of known targets of the gene products under study [12,27]. Primers were designed and checked by BLAST searching to avoid redundancy as described [7]. The gene-oligo array consisted of 26,090 70-mer oligonucleotides from the *Arabidopsis *genome oligo set Version 1.0 [41], arrayed and hybridized as previously described [7].

### Hybridization to microarrays

Samples were dissolved in Tris-EDTA (TE) buffer and 50 to 60 μg aliquots were subjected to digestion by addition of 200 units of restriction endonuclease for 3 to 4 h. *Msp*I endonuclease was obtained from New England Biolabs (New England Biolabs, Ipswich, MA, USA). Digested DNAs were size-fractionated on 5% to 30% sucrose gradients as described [42]. Aliquots of DNA fractions were examined by agarose gel electrophoresis to verify DNA fragment size and concentration. Fractions in the <2.5 kb range were pooled, precipitated by addition of ethanol, and fluorescently labeled with either Cy3 or Cy5-dCTP (Amersham, Piscataway, NJ, USA) by random priming (Invitrogen) as described [42]. Oppositely labeled samples from mutant and wild-type were mixed together and hybridized to microarrays on glass slides and processed as described [42].

### Data processing

Slides were scanned using a GenePix 4000 fluorescent scanner (Axon Instruments Inc, Union City, CA, USA). For each mutant comparison, three to four biological replicates were performed, all with dye-swaps. A lowess normalization was applied to the gene-oligo array to correct for non-linearity in this dataset. Methylation profiles were analyzed and *p* values assigned using Cyber-T microarray analysis software, which applies a Bayesian T-statistic method [21]. The data-versus-model weighting factor was adjusted to 8 for the random-PCR array and to 6 for the gene-oligo array. A window size of 161 was used for the random-PCR array and 201 for the gene-oligo array. Bayesian-derived *p* values were adjusted for multiple hypotheses testing using a Bonferroni correction (*p* = 0.05) for the random-PCR array and a false discovery rate of *p* = 0.05 for the gene-oligo array. Note that the use of statistical criteria to delineate targets results in greatly reduced sensitivity of the gene-oligo array relative to the much smaller random-PCR array. An additional criterion for significance was implemented using 'self versus self' control experiments to assess experimental variation within the system. Accordingly, a lower-bound threshold for the log_2 _methylation ratios (cy3/cy5) was defined as 3 standard deviations for the random-PCR array (4 for the gene-oligo array) from the corrected mean of the distributions of log_2_-transformed ratios.

### Analysis of methylation profiling data

To facilitate comparison of datasets, we implemented a relational database (mysql) with a web browser display (Methprof [25]). Methprof has utilities for processing raw data and for statistical analysis by CyberT [21]. Methprof displays positive hits based on CyberT analysis for individual and combined datasets, together with a graphical chromosomal map of all the loci. Each hit in a Methprof table links to annotation data and displays user-provided descriptions, the number and identity of datasets in which it is positive, and whether the hit is hypo- or hyper-methylated.

In addition, a Javascript program (Region Viewer, developed by us) was implemented to display annotation and restriction site data for loci on the PCR-based array, and Methprof was adapted to display similar information for the oligo-based array. For each locus, a 'neighborhood' centered on a locus was defined such that blockage of a methyl-sensitive restriction site anywhere in the region could increase a fragment from less than to greater than the 2.5 kb cutoff. The blocked site was inferred as that most likely to have caused the depletion from the <2.5 kb fraction, ignoring ambiguous cases. Gene information was parsed from Genbank entries. Repeat information was generated using the program Censor4.1 [43] on the *A. thaliana *repeat library athrep.ref [23]. Repeat information was also obtained by BLASTN searching of an *A. thaliana *library of consensus sequences generated by the RECON program (Zhirong Bao, personal communication) [24]. Data and maps used in this study are available for querying, browsing or downloading using Methprof [25], and all raw data can be downloaded from the GEO database under Accession number GSE3109 [44].

## Figures and Tables

**Table 1:** Loci scored as CNG methylation targets in mutants on the random-PCR array

Genomic location*	Gene ID	TIGR designation					*drm1*
						*drm1*	*drm2*
			*cmt3*	*kyp*	*ago4*	*drm2*	*cmt3*
Random loci							
1:14147723-14148423		Non-LTR retrotransposon family (LINE)	-	-			-
3:18799179-18799859	At3g50620	Intergenic	-	-			-
4:1813417-1814107		Mutator-like transposase family	-	-			-
2:8651310-8652034	At2g20020	Expressed protein	-		-		
2:11049695-11050396	At2g25900	Zinc finger (CCCH-type) family protein				-	-
3:19989359-19990040	At3g53960	Proton-dependent oligopeptide transport	-				
2:5627581-5628302		non-LTR retrotransposon family (LINE)		-			
1:8273192-8273908	At1g23320	Alliinase family protein contains Pfam					-
1:22107946-22108642	At1g60020	Copia-like retrotransposon family					-
2:7532595-7533279	At2g17305	Hypothetical protein					-
2:13188318-13189035	At2g30970	Aspartate aminotransferase, mitochondrial				-	

*Chromosome number:span of fragment in TIGR map April 2004. Dashes indicate loss of methylation and blank spaces indicate no significant change.

**Table 2:** CNG methylation changes within transposable elements

Mutant	Total targets	Transposon targets*	*p* value^†^
**Random-PCR array**			
All loci	597	12	
*cmt3*	5	2	0.01
*kyp*	4	3	<0.0001
*ago4*	1	0	1
*drm1/2*	2	0	1
*drm1/2 cmt3*	7	3	0.001
**Gene-oligo array**^**‡**^			
All unambiguous loci	6,950	907 (13%)	
*cmt3 *targets	164	104 (63%)	<0.0001
*kyp *targets	38	26 (68%)	<0.0001
*ago4 *targets	0	0	-
*drm1/2 *targets	6	4 (67%)	0.005
*drm1/2 cmt3 *targets	103	67 (65%)	<0.0001

*Total number of possible transposable element hits among the 597 loci on the random-PCR array or among the 26,090 loci on the gene-oligo array. ^†^Using Fisher's exact test. ^‡^All numbers represent only those loci for which the *Msp*I cut site responsible for differential fractionation could be unambiguously determined.

**Table 3:** Percent DNA methylation of elements in mutant lines^1^

		Genotype																							
		
		L*er* (wt)			Clk-st (wt)			Ws (wt)			*cmt3*			*kyp*			*ago4*			*drm1/2*			*drm1/2 cmt3*		
Locus	Size	CG	CNG	CNN	CG	CNG	CNN	CG	CNG	CNN	CG	CNG	CNN	CG	CNG	CNN	CG	CNG	CNN	CG	CNG	CNN	CG	CNG	CNN
MU-4802^2^	4.8	84	34	19	75	28	13	79	25	9	84	1	4.8	84	0.5	1	64	30	19	74	27	9.4	79	0.5	2.8
TA11-4217^3^	6	-	-	-	81	62	12	88	77	12	75	7.5	10	80	42	13	82	65	12	81	60	5.5	9.6	0	0.2
SIN3-5300^4^	0.2	80	78	38	80	70	59	88	72	58	90	10	6.8	83	73	54	58	2.5	0	58	1.7	0	15	2.5	0
MU-PCR^5^	>7	88	47	8	80	43	7.5	-	-	-	63	1.1	4.8	74	16	7	85	43	8	81	54	7.5	89	0.5	4.9
ATSN1^6^	0.2				**75**	**70**	**24**				**49**	**8.7**	**8.7**	**82**	**53**	**29**	**42**	**14**	**0.8**	**53**	**30**	**3.3**	**28**	**0**	**0.2**
SUP^7^	1.2				**16**	**55**	**16**				**8.9**	**0**	**5.7**	**5.9**	**1.7**	**1.9**	**16**	**20**	**3.6**	**14**	**39**	**8.7**	**10**	**0**	**0**
MEA-ISR^8^	1.3				**95**	**58**	**26**	**87**	**47**	**18**	**89**	**13**	**13**	**92**	**25**	**17**	**81**	**0**	**0.7**	**87**	**0**	**0**	**87**	**0**	**0**
FWA^9^	0.5				**84**	**16**	**6.8**	**89**	**18**	**4.2**	**93**	**2.9**	**6.4**				**87**	**2.5**	**0.7**	**81**	**0**	**0**	**88**	**0**	**0**
NOS-PRO^10^	0.4	71*	45*	38*							**54**	**19**	**24**				**77**	**21**	**10**	**77**	**23**	**3.2**	**61**	**1.3**	**0.3**
SIN3-11193^11^	0.2	N/A	N/A	N/A	N/A	N/A	N/A	91	90	56	N/A	N/A	N/A	N/A	N/A	N/A	N/A	N/A	N/A	81	2.5	0.3	N/A	N/A	N/A
229R1^12^	?	-	-	-	93	58	13	93	58	7	83	1	1.7	88	17	6.3	88	54	5.1	87	49	1.9	76	0.5	0.5
229R2^12^	?	-	-	-	-	-	-	94	48	2.1	-	-	-	-	-	-	-	-	-	73	0	0	-	-	-

^1^The fraction methylated reported in Figure 5 is the ratio of mutant to wild-type. For example, the fraction of TA11-4217 that is methylated in *ago4 *is 65% (*ago4*)/62% (Clk-st) = 1.05. Numbers in bold are from previously published work. All loci except for 229R1 and 229R2 were used in the scatter plots in Figure 5. We determined the size of each element based on the Repbase consensus sequence, except for published examples, in which we used the information provided in the references below. ^2^MU-4802 is an isolated Mutator-like element annotated as At1g17275. ^3^TA11-4217 is an isolated LINE element annotated as At1g29650 with very close homology to TA11. ^4^SIN3-5300 is a SINE3 element located between At3g60130 and At3g60140. ^5^MU-PCR is a complex locus of multiple transposable elements inserted into one another present at the hk4S heterochromatic knob on chromosome 4 in Ws and in pericentric heterochromatin on chromosome 4 in L*er* [19]. ^6^AtSN1 is a SINE1 element [33]. Bisulfite sequencing data derived from [12]. ^7^SUP is the *Arabidopsis SUPERMAN *gene [45]. Bisulfite sequencing data derived from [12,16,27,46]. ^8^MEA-ISR is a locus composed of tandem direct repeats located downstream of the *Arabidopsis MEDEA *gene [46]. Bisulfite sequencing data derived from [12,46]. ^9^FWA is the *Arabidopsis FWA *gene [47]. Bisulfite sequencing data derived from [11,46]. ^10^NOS-PRO is a transgenic nopaline synthase promoter [32]. Bisulfite sequencing data derived from [10,29]. ^11^SIN3-11193 is a SINE3 element located between At3g22060 and At3g22070. ^12^229R1 and 229R2 are intergenic sequences within 2 kb of one another located between At1g36940 and At1g36950. Dashes indicate that it is not done; N/A, sequence absent in L*er*; asterisks indicate that the numbers are for the transgenic NOS-PRO line, Col ecotype; question marks indicate that the size of the methylated sequence cannot be accurately predicted.

**Table 4:** Primers used for bisulfite sequencing

SIN3-11193 (A011193)	G->A: 5'-CCTCCTTCGTTGACCTGTCTTCATCGCAATGACTCAGCATAG-3' C->T: 5'- GTCTTCTAATCAAGTTTAGTTATGTTAATGTTTTTGGATAGAAC-3'
SIN3-5300 (A005300)	G->A: 5'-TTCATTTGTTACCTACTATCATTTTCAAGAACGAAACAATG-3' C->T: 5'-TAGTAGTTGTTCTCATCTTGTTTTTGGCAACTGGACGTGTC-3'
229R1 (A000229)	G->A:5'- CACCATGTTCTAGCCCTTGTTCGGTCGTCGTTCCTTCCGTGG-3' C->T: 5'- AAAAGAAAGGCGTCGTGGAATCACCACTAGCTACAACCGC-3'
229R2 (A000229)	G->A:5'- TTAGAGCTTGTTTTCATTACCTTCTTCACACAACCTCCAAG-3' C->T: 5'- TTTCAGGGTATCATGGTTCTCGACAAAGTAGGGTTATTATC-3'
TA11-4217 (A004217)	G->A:5'- CAACATAAGATTGTAGCCTTCCATCCTTGACCACGCTTTG-3' C->T: 5'- TCTTAAGATAGGAGATGATGTGTAGGAATGGTTTCTGGCAC-3'
MU-4802 (A004802)	G->A:5'- AGCCATTATCATGTCCATCTGATCCTTCTACATGCCCTTG-3' C->T: 5'- TATGTGAACGACTCATACACAAGAAATAGGTGGCGAGAAAC-3'
MU-PCR (57802433)	G->A:5'- CACCAGCTCGAACACCACCAACAGATTCCTTGTAAATCTG-3' C->T: 5'- GATGGAGCGAGTGACGGGGATGAAGAGTCTAGTGTGTGCAC-3'

To amplify bisulfite-treated DNA, primers were synthesized with G→A (first sequence of the pair or C→T (second sequence), except for CGs and CNGs, which were synthesized with G→R or C→Y, respectively.
